# Facial Paralysis Treatment Using Selective Neurectomy: A Comprehensive Review

**DOI:** 10.7759/cureus.51809

**Published:** 2024-01-07

**Authors:** Bader Fatani, Hissah S Alshalawi, Lujain A Alsuhaibani, Turky M Alrasheed, Ghaida A Alislimah, Afraa Al-Safadi

**Affiliations:** 1 Dentistry, College of Dentistry, King Saud University, Riyadh, SAU; 2 Surgery and Pharmacy, King Khaled University Hospital, King Saud University Medical City, Riyadh, SAU

**Keywords:** complications, synkinesis, selective neurectomy, facial nerve paralysis, facial paralysis

## Abstract

Facial paralysis can affect patients undergoing full mouth rehabilitation, regardless of what caused their paralysis. A procedure known as modified selective neurectomy of the facial nerve can enhance the movement of facial muscles in individuals with facial synkinesis safely and effectively. This approach is proposed as an alternative to more invasive surgical options when used independently as a treatment for incomplete facial palsy. Selective neurectomy offers a promising surgical option for managing nonflaccid facial paralysis and synkinesis, enhancing patients' quality of life. However, treatment plans should be individually tailored considering the complexity of facial paralysis and the unique needs of each patient, taking into account the timing and type of treatment. The objective of this review is to explore the use of selective neurectomy as a treatment for facial paralysis based on previously published papers.

## Introduction and background

Facial paralysis can be attributed to known and unknown factors, such as injuries, growths, viral infections, or medical procedures [1]. Coping with long-term paralysis can be challenging and have adverse effects on the patient's mental health and social life. Nevertheless, surgical treatments can enhance their quality of life [2]. Social interactions can pose difficulties for individuals with facial nerve paralysis, as facial expressions are essential nonverbal signals that convey meaning, intentions, and emotions [3]. Patients in recovery from facial palsy may encounter issues with facial expression, disrupted resting facial posture, and unpredictable psychological outcomes due to persistent synkinesis [4]. Approximately one in 10 to one in five individuals with Bell's palsy will experience synkinesis, though generally not severely [5]. The approach to treating facial nerve paralysis that cannot be directly repaired or with grafting usually depends on the duration of the problem. For shorter periods, nerve transfers may offer a potential solution. However, in cases of long-standing issues, recruiting new neuromuscular units through a local or free tissue transfer may be necessary [6]. Individuals with facial paralysis can benefit from receiving facial rehabilitation irrespective of the underlying cause. After undergoing dynamic facial reanimation, facial rehabilitation is a critical aspect of postoperative management [7]. A procedure referred to as modified selective neurectomy of the facial nerve can reliably and effectively improve the movement of facial muscles in individuals with facial synkinesis. As an alternative treatment for incomplete facial palsy, this approach is recommended when used as a standalone option, instead of more invasive surgical choices [8]. This review aims to explore the use of selective neurectomy in the treatment of facial paralysis.

## Review

Methodology

This review evaluated published papers that discussed the use of selective neurectomy in the treatment of facial paralysis. PubMed and Google Scholar were utilized to collect the most relevant papers by using keywords like selective neurectomy and facial paralysis. By applying this method, all papers related to the use of selective neurectomy in facial paralysis treatment were obtained. Only relevant papers that met the inclusion criteria were included, while studies that fell outside the scope were excluded. The inclusion criteria included studies that discussed facial paralysis, synkinesis, selective neurectomy, outcomes, and complications. Studies that had inadequate methodology, outdated information, and insufficient data were excluded. The initial screening process identified 152 papers, and after applying the inclusion criteria, 46 relevant papers were chosen for the review.

Facial paralysis and synkinesis

Facial nerve disorders commonly result in initial complete paralysis, but the extent of recovery varies depending on the underlying cause [8]. Synkinesis can occur as a result of abnormal reinnervation after facial paralysis, where both correct and incorrect muscles receive nerve signals [8]. This can obstruct the activation of key smile muscles and lead to a downward and sideways pull on the oral commissure, reducing the visibility of the upper and lower teeth [8]. The goal of treatment is to improve overall facial appearance and function, with modified selective neurectomy being a technique that removes unwanted facial movements and enhances natural smiles [9]. Facial nerve dysfunction can significantly impact functional abilities, appearance, and psychological well-being, and can be caused by various factors such as Bell's palsy, injuries, infections, or autoimmune diseases [10]. The House-Brackmann Facial Nerve Grading System is commonly used to measure patient outcomes, but alternative tools like the eFACE instrument allow for more detailed evaluations [10]. Bell's palsy is the most frequent cause of facial nerve paralysis, and patient outcomes can range from complete ongoing flaccid paralysis to complete restoration of function [11,12]. Facial hyperkinesia refers to uncontrollable facial muscle contractions and should be differentiated from neurotic tics. Facial hyperkinesia spasms can persist even during sleep or anesthesia and cause distressing symptoms [13].

Synkinesis, which is the involuntary movement of one part of the face when another part is intentionally moved, is commonly observed in individuals who have previously experienced flaccid facial paralysis [10]. It is believed to occur due to aberrant regeneration, ephatic transmission, and hyperexcitability at the level of the facial nucleus [10]. Synkinesis can have a negative impact on a patient's quality of life and can be treated with botulinum toxin injections and neuromuscular retraining. Surgery options include selective myectomy or neurectomy, cross-facial nerve grafting, nerve substitution, and free gracilis muscle transfer [14]. The occurrence of synkinesis in patients with long-lasting facial paralysis is reported to be around 55%, but there is still limited understanding of this debilitating situation, resulting in restricted abilities and reduced quality of life [15]. Synkinesis usually develops within three to four months after facial paralysis onset, and the most widely accepted theory for its development is aberrant regeneration, where nerve injuries cause improper muscle movement and contractions [16]. Moderate to severe synkinesis can cause aesthetic and functional consequences like facial asymmetry, narrowing of the eye aperture, and difficulties in food manipulation. Dysfunctional synkinetic movement of muscles around the lips and cheeks can pull the oral commissure downwards and sideways, leading to a frozen or frowning smile, decreased teeth show, and psychological impacts on well-being [16]. The management of traumatic facial nerve injury poses a dilemma as to whether to opt for surgery or not [17]. Palliative surgery aims to provide relief for individuals with facial paralysis, serving as a temporary solution until nerve recovery occurs, or as a conclusive intervention in cases involving permanent loss of muscle functionality [18].

Selective neurectomy

Facial paralysis is a complex condition that results in the loss of voluntary muscle movement in the face and can have various causes such as trauma, infectious diseases, Bell's palsy, and acoustic neuroma growth [16,19]. One challenging aspect of facial paralysis is synkinesis, where intentional movement in one area of the face leads to unintentional movement in another, causing facial asymmetry and dysfunction that significantly impacts a person's quality of life [10,16]. To address these challenges, selective neurectomy has emerged as a viable surgical treatment option for facial paralysis, particularly when synkinesis is present [9]. This procedure involves identifying and removing specific nerve-muscle connections responsible for involuntary facial movements, aiming to reduce hypertonicity and involuntary movements associated with nonflaccid facial paralysis [9,19]. Selective neurectomy can complement other management approaches like neuromuscular retraining, Botox, selective myectomy, and reanimation procedures, with several studies demonstrating improved facial symmetry, function, and quality of life [14]. Botulinum toxin type A (BTA) is the preferred treatment for facial synkinesis, playing an essential role in the therapeutic approach for patients with this condition [20].

The process of selective neurectomy involves a comprehensive preoperative assessment, including patient history, physical examination, and tools like eFACE or Sunnybrook Facial Grading system. During surgery, the nerve branches causing abnormal muscle contractions are selectively cut, eliminating antagonistic movements characteristic of nonflaccid facial paralysis [9,19]. Evidence suggests promising results with selective neurectomy, reducing involuntary facial movements, and hypertonicity, and improving overall facial function and appearance [9,19]. However, further research is needed to understand the long-term outcomes and potential complications associated with this procedure [19].

In addition to traditional selective neurectomy, a modified technique has shown promise in treating synkinesis following facial palsy. This modified method involves careful dissection and stimulation of specific facial nerve branches, followed by the severing of branches causing undesired movements [16]. Postoperative care usually includes neuromuscular retraining and additional treatments as necessary [16]. Combining selective neurectomy with other treatments like masseteric-to-facial nerve transfer has shown potential in improving patient outcomes, as indicated by improvements in eFACE scores [10]. 

Selective neurectomy offers a promising surgical option for managing nonflaccid facial paralysis and synkinesis, enhancing patients' quality of life. However, treatment plans should be individually tailored considering the complexity of facial paralysis and the unique needs of each patient, taking into account the timing and type of treatment [19]. The modified selective neurectomy procedure targeting the buccal and cervical branches of the facial nerve has proven to be a successful and lasting solution for smile dysfunction in individuals experiencing synkinesis after facial paralysis [8].

Indication and contraindication 

Selective neurectomy is a surgical treatment for nonflaccid facial paralysis that primarily addresses synkinesis and facial hypertonicity. It is recommended for patients experiencing persistent synkinesis or hypertonicity that greatly impacts their quality of life, especially when non-surgical interventions like physical therapy or botulinum toxin injections are not effective. However, this procedure is not suitable for patients with poor overall health that may complicate the surgery, those with incomplete facial paralysis expecting a spontaneous recovery, individuals unlikely to adhere to postoperative care and rehabilitation, and cases without significant synkinesis or hypertonicity. Therefore, a careful selection process for patients, considering the potential benefits and risks, is crucial for successful outcomes [10,16,19].

Surgical approach

The surgical procedure used for facial hyperkinesis was first described in 1972. It involves removing extratemporal nerve branches that cause excessive facial movements while preserving innervations to avoid facial paralysis and its complications [13]. After exposing the nerve trunk and bifurcation, the upper zygomaticotemporal division is identified, taking care to preserve the frontal ramus near the superficial temporal artery [13]. The nerve exposure continues until all first and second-order branches of the superior division are visible [13]. Stimulation is then performed on the exposed nerve branches using bipolar forceps and an appropriate voltage level that elicits a visible response from the corresponding facial muscles when touching the nerve itself, not the surrounding tissue. The recognized facial nerve branches are then coagulated, clipped, and cut as proximal as possible. The distal stump of the nerve branch is elongated, coagulated, and clipped after being pulled and grasped with a hemostat. Resection is then carried out on the elongated segment of the nerve. To achieve a lasting satisfactory outcome, all nerve fibers innervating the orbicularis oculi and superior buccal branches are excised in cases of hemifacial spasm and blepharospasm [13].

Selective neurectomy is considered successful when stimulation of the main trunk fails to produce any movement in the orbicularis oculi muscle, but normal contractions are observed in the frontal area and the lower part of the face. If the spasm affects the lower lip, chin, and platysma, resection should be performed on the ramus colli and one or two of the three main divisions of the ramus marginalis mandibulae. The authors briefly discussed four ramification patterns to clarify the extent of selective neurectomy, based on their work with 40 cases of blepharospasm, according to Davis et al. Type I ramification pattern, found in 20% of cases, is characterized by the absence of anastomosis between the revealed branches of the facial nerve. In this pattern, the buccal ramus arises from either the superior or inferior division of the facial nerve. Excision includes all branches related to the orbicularis oculi muscles, as well as half of those related to the levator labii superioris, zygomaticus, and risorius muscles. When different rami have a common origin, a close excision is performed proximal to the main superior division of the facial nerve [13].

Type II ramification pattern is present in 37.5% of patients, where the lowest orbicular branch shares a common origin with the superior buccal branch. In selective neurectomy of the superior buccal branch, the exposure needs to be continued distally until the corresponding ramification of the nerve is found. If there is no natural splitting of nerve fibers, the superior half of the exposed nerve is excised using a watchmaker's forceps and nerve scissors. In this situation, the application of silver clips or coagulation of the proximal and distal stumps is not possible. Dissection is performed in the frontal region where the orbicular and frontal fibers run within the same nerve branch. Type III ramification pattern, present in 10% of patients, is characterized by buccal branches originating from both the superior and inferior divisions of the facial nerve. Selective nerve excision is necessary to fully expose the superior and inferior divisions of the facial nerve. In cases where there is no natural nerve branching, a precise and clear dissection is performed to separate the associated fibers. Magnifying loops can be helpful in identifying the division between different fiber bundles of the same nerve branch [13].

Type IV ramification pattern, found in 15% of patients, involves a sling-like anastomosis between the superior division and buccal branches of the facial nerve. Type V and type VI patterns also feature sling-like anastomosis but within the first-order branches of the superior division of the facial nerve. These patterns are observed in 15% and 25% of patients, respectively. In patients with hemifacial spasms, preservation should be considered for some superior buccal nerve fibers to maintain symmetry in smiling and laughing. After facial paralysis, associated movements occur, and patients should exercise the majority of fibers in the superior buccal region and orbicularis oculi muscle to reduce the tendency for lid closure when stimulating facial muscles [13].

The extent of nerve excision is determined by the severity of facial movements and the approximate amount of postoperative return in facial function after regeneration. In all cases, total denervation of the orbicularis oculi muscle and partial denervation of the midfacial region is required. For hemifacial spasms, branches that contribute to "opening up" the face, such as those that depress the corner of the mouth and elevate the eyebrow, should be preserved. If spasm is involved, resection of portions of lower branches is necessary. Preservation of the frontalis muscle is important to allow reinnervation of the eye. The decision on the amount of resectable nerve tissue depends on the surgeon's experience with various facial nerve problems. It is surprising how minimal innervation is needed for normal facial movement if the existing nerve fibers are accurately distributed. This knowledge is crucial for the development of direct regeneration techniques in facial nerve grafting [13].

Reinnervation following selective neurectomy for hyperkinesia can occur in two ways, as observed during revision surgery. Firstly, axons can regenerate through the surgical region despite proximal stump clipping, cautery, and resection of 3-4 cm of nerve. This process, known as “open-field” regeneration, can cover long distances. It is notable that the nerve can traverse the previous surgical field and replace small branches that were absent in the initial operation, as these branches are of appreciable size and can be directly dissected from scar tissue containing surgical clips. Secondly, vertical anastomotic branches can be observed medially, near the midline, connecting midfacial and orbital musculature to unresected buccal and mandibular branches. These pathways may have existed prior to surgery and carry a small number of axons. If stimulation of the branches to be conserved during the initial operation does not produce movement in the orbital musculature, vertical anastomotic branches could play a role in reinnervation [13].

These small branches have a significant role in the postoperative period through collateral support. Intact axons that maintain functional connections to their normal motor units may generate sprouts that grow and innervate previously denervated motor units. The extent to which this occurs in facial musculature is still uncertain. Motor units that require new innervation or are still innervated can hypertrophy, providing another mechanism for previously insignificant neural pathways to restore function. The presence of distal vertical anastomotic branches can be observed during revision surgery by activating the lower buccal and mandibular branches after removing the recurrent midfacial and orbital branches, resulting in continued contractions of the upper lip and eye. Based on experience, these fibers need to be dissected along the zygoma near the midline. Two additional approaches to reinnervation should be mentioned. First, each facial nerve may innervate some musculature on the opposite side. Electroneurography studies have shown potentials up to 2.5 cm beyond the midline after activating the contralateral facial nerve trunk. In cases of parotid malignancy with facial nerve resection, reinnervation can be hindered by contralateral facial nerve block. Contralateral innervation is mostly limited to the lower part of the face, particularly the orbicularis oris and mentalis muscles. It is uncertain whether this plays a significant role in recovering orbital muscle function after hyperkinesia surgery. Second, there is evidence from clinical and basic research suggesting that the trigeminal nerve may supply the facial muscles after facial nerve resection. Most of the cases discussed in this paper involve parotid malignancy, and direct trauma to the masticatory musculature can result in the regeneration of trigeminal nerve axons that find their way into perineurial tubules, reaching the facial muscles [13].

The authors' position is that the reinnervation process resulting from selective neurectomy for hyperkinetic cases does not involve trigeminal-facial communications, hence it does not cause trauma to muscles innervated by the trigeminal nerve [13]. When revision surgery is required, a flap is raised after reopening the previous incision over the parotid gland and anterior to it to access the peripheral nerve branches [13]. The branches responsible for recurring twitches are identified through electrical stimulation and palpation, potentially using silver clips [13]. The involved nerves are excised with small blocks of adjacent parotid tissue, extending the excision medially to the masseter muscle. No facial movement occurs when stimulation is applied to the excision area. Eye closure controlled by the buccal or mandibular branches can be observed through stimulation in a more medial and inferior direction. Careful dissection of soft tissue along the orbital rim and zygomatic arch is crucial to locating the peripheral branches that innervate the eye and midface [13]. These branches are cauterized until the desired movement, such as brow elevation and downward movement of the corner of the mouth, is achieved and observed through electrical activation anywhere on the face. Finally, as in the primary surgery, the wound is closed after drainage [13]. For complete unilateral facial paralysis, an effective and safe procedure called end-to-trunk masseteric to facial nerve transfer has been performed, resulting in a natural-looking smile [21]. Decompressing the facial nerve did not provide any advantages in cases of traumatic facial paralysis resulting from closed-head injuries [22]. Assessing nerve excitability in peripheral branches has proven valuable in predicting outcomes and identifying patients for whom surgical intervention may not be necessary [22].

Outcomes 

Facial paralysis can have a profound impact on an individual's quality of life, hindering their ability to communicate, speak, eat, and perform daily activities. To address this, selective neurectomy is a surgical procedure used to treat specific types of facial paralysis, aiming to alleviate symptoms and improve facial function [14]. The results of this treatment option demonstrate significant enhancements in facial symmetry, facial function, and overall quality of life [14]. In a retrospective review of patients who underwent modified selective neurectomy, there was a statistically significant improvement in various aspects of facial function, such as oral commissure movement with a smile, lower lip movement, nasolabial fold depth at rest, dynamic and static scores, lower and mid-face scores, and smile score [8]. Nevertheless, there remains a substantial lack of knowledge regarding this treatment approach, making extensive, well-designed prospective research comparing it to alternative options essential [14]. Patients with post-facial paralysis synkinesis experienced a better quality of life after undergoing a modified selective neurectomy [23]. Furthermore, hemifacial spasms disappeared in 65.6% of patients following selective facial neurectomy [24].

Complications 

Selective neurectomy, a treatment for facial paralysis, is generally regarded as a safe and effective procedure, with no significant complications observed during or after treatment. The most common complications are hemostasis and brief oral incapacity. In some cases, a second treatment involving the resection of additional nerve branches may be necessary for optimal outcomes [8]. A retrospective analysis by Azizzadeh et al. was carried out on a group of patients who received a modified selective neurectomy procedure on the buccal and cervical branches of the facial nerve. This procedure was performed by a single surgeon over a period of four and a half years. The study evaluated the changes in House-Brackmann facial grading scores, electronic clinician-graded facial function scale, and dosages of ona-botulinumtoxin A (botulinum toxin type A) before and after the surgery. A total of 63 patients received a modified selective neurectomy procedure, with no occurrence of significant complications. The revision rate was found to be 17%, while seven patients (11%) experienced temporary oral incompetence after the surgery [8]. Furthermore, seven patients reported temporary oral incompetence after the procedure [9]. Overall, selective neurectomy is an effective approach for treating facial paralysis, as it has the potential to restore facial function and improve the quality of life for affected individuals. The success and effectiveness of the procedure can be evaluated on a case-by-case basis, taking into account individual factors and careful assessment [8]. In cases of muscle action recurrence after denervation, symmetrical paralysis can result from a neurectomy on the unaffected side following unilateral forehead paralysis caused by a facelift. However, gradual motion spread in the denervated paramedian supraorbital area occurs within two months. This is because all expression muscles originate from the same mesodermal mass and the peripheral anatomy of the facial nerve. Regeneration was found to take 12 to 18 months, and the nasolabial area exhibited the highest recurrence rate [25,26].

Additional findings

In a previous study by Biglioli et al., the authors suggest separating the neural signals that control the orbicularis oculi muscles from those that control the zygomatic muscular complex as a treatment for synkinesis related to eyelid closure and smiling. In 83.33% of cases, synkinesis was entirely resolved, and all patients experienced a noticeable improvement in their facial movement. The use of neurorrhaphy on the masseteric nerve and the central branch of the facial nerve seems to lead to positive outcomes [27]. Biglioli et al. also explained that in cases of recent facial paralysis, where muscle spasms are still observable using electromyography (EMG), facial reanimation can be achieved by introducing new neural signals to the muscles. However, if a significant amount of time has passed, the mimetic muscles may suffer from irreversible atrophy, rendering a new neural stimulus ineffective. in such situations, the facial function can be restored by surgically transferring free flaps to the face or repositioning masticatory muscles to reinstate major movements like eyelid closure and smiling [28]. Moreover, the author explained that the utilization of two motor nerves, the masseteric nerve and a portion of the hypoglossus nerve fibers, along with a qualitative neural source (the contralateral facial nerve connected through two cross-face nerve grafts), seems to enhance facial movement restoration after paralysis, overcoming previous skepticism regarding the ability of patients to utilize various nerves for facial expressions. As a matter of fact, there was a considerable improvement in movement. When it comes to smiling based on emotions and blinking, the use of a two-step cross-face nerve grafting procedure appears to provide much better assurance [29]. 

The objective of facial reanimation surgery is to reinstate significant movement in the face. The aim should be to establish clinical practice guidelines that prioritize quality in order to enhance patient care. It is crucial for facial reanimation surgeons to take the lead in using consistent outcome measures to report their findings, in order to initiate this process [30]. Reconstructive surgeons face a complex task in achieving facial animation. There is a wide array of surgical techniques and modifications available, and the choice of approach depends on factors such as the specific type and timing of facial palsy, the patient's age, prognosis, and overall health condition. It is crucial for surgeons to have a comprehensive understanding of all available approaches to effectively plan facial reanimation surgery [31]. The popularity of utilizing the masseteric nerve branch for facial reanimation is increasing. Its versatility, anatomical location, ease of dissection, low risk, and potential for motor neural input make it an excellent choice for various reanimating techniques. The selection of the appropriate nerve should consider factors such as the type and timing of facial paralysis, the patient's age, prognosis, and personal preferences. Having a comprehensive understanding of the advantages and potential disadvantages of using this nerve is vital knowledge for surgeons specializing in facial reanimation [32]. Terzis et al. concluded in their study that a highly effective surgical approach for addressing synkinesis after facial palsy is cross-facial nerve grafting, along with secondary microcoaptations and botulinum toxin injections. This method significantly improves facial function and symmetry. In addition, facial neuromuscular re-education plays a significant role in the treatment process [33]. Markey et al. on the other hand explained in their study that the primary treatment approach for facial synkinesis remains physical therapy and neuromodulation using botulinum toxin. The use of neurotoxin to treat muscles such as the orbicularis oculi, mentalis, and platysma is well-documented. Achieving a more balanced smile can also be accomplished by weakening the ipsilateral depressor anguli oris and contralateral depressor labi inferioris muscles. Additionally, innovative surgical methods for selectively eliminating specific facial muscles have been recently reported [34]. Moreover, depressor anguli oris resection is a low-risk procedure that often leads to enhanced smile movement, balanced smile appearance, and overall improved quality of life in individuals with nonflaccid facial palsy [35]. Krag et al.'s study concluded that the use of a depressor anguli oris muscle block resulted in improved symmetry at rest, as well as the angle and visibility of teeth during smiling. This highlights the inhibitory mimetic function of an overactive depressor anguli oris muscle in synkinesis. It serves as an important tool for diagnosing and communicating hypertonicity in the depressor anguli oris muscle and suggests the potential benefits of future depressor anguli oris myectomy [36]. In addition, the Halani et al.'s study showed that the effectiveness of depressor anguli oris myectomy in addressing asymmetry in patients with synkinesis necessitates additional technical improvements in depressor anguli oris transfers or alternative methods to enhance lower lip depression in this specific patient subgroup [37].

The depressor muscles of the lower lip play a crucial role in achieving a complete smile with dentures and expressing facial emotions. To restore the function of these muscles, both static and dynamic techniques can be employed. Static techniques aim to achieve symmetry by weakening the normal side through methods such as botulinum toxin injection, depressor labi inferioris myectomy, and marginal mandibular nerve neurectomy. Another approach is to create static slings and perform tightening procedures on the affected side. On the other hand, dynamic techniques focus on restoring functionality by reanimating the affected depressor complex using nerve transfers, muscle transfers, and direct muscle neurotization [38]. Lengthening myoplasty and temporalis muscle transposition are alternative choices for patients who are not suitable for neurotization using the facial nerve. To achieve both a genuine, natural smile and movement in the facial muscles, the preferred surgical approach for the authors is the utilization of free microneurovascular muscle transfer, with neurotization from the healthy facial nerve on the opposite side [39]. The introduction of microsurgery revolutionized the restoration of facial movement in individuals with facial paralysis through the use of techniques such as cross-facial nerve grafts, nerve transfers, and free muscle transplantation. Currently, nerve transfers are the cornerstone of facial reanimation, particularly in situations where reconstructing the affected facial nerve is not possible. The appropriateness of each nerve transfer procedure depends on factors such as the specific type of facial paralysis, the time that has passed since the injury, as well as the patient's age and overall health. It is crucial to select a motor nerve that can produce strong muscle contractions and allow the patient to control their facial movements [40]. Free tissue transfer, on the other hand, provides the opportunity for synchronized and natural movement. However, it does require a longer healing period compared to regional muscle transfer. The decision on which approach to use is made collaboratively by the physician and the patient, considering their preferences and biases. For uncomplicated facial paralysis without skin or soft tissue deficiencies, our preferred choice is muscle-alone free tissue transfer. In more complex cases involving facial paralysis with skin or soft tissue deficits resulting from tumor removal, a combination of muscle and other tissues, often including a skin flap, is another preferred option [41]. Creating a natural and symmetrical smile with normal resting muscle tone is a crucial aspect of addressing the functional limitations and social challenges associated with facial paralysis. While multiple surgical techniques have been described, the most commonly used approach involves a two-stage method, starting with a cross-facial nerve graft followed by a free functional muscle transfer. However, for certain patients, a single-stage reconstruction using the motor nerve to the masseter as the donor nerve is considered superior to the two-stage repair. The gracilis muscle is frequently utilized for reconstruction due to its consistent anatomy, ease of dissection, and minimal complications at the donor site [42].

Park et al.'s study involved the examination of 122 patients who underwent selective neurectomy. Before the surgery, the patients were given nine questionnaires that aimed to identify their two primary concerns or complaints regarding the treatment. After the surgery, the patients were evaluated for facial tightness, limited mouth movement, and narrowing of the eyelid aperture. The authors concluded that selective neurectomy can effectively address concerns related to facial tightness and narrowing of the eyelid aperture. It can significantly enhance the upward movement of the mouth corners, although the improvement in horizontal angles may not be as optimal [43]. Chuang et al.'s study aimed to present proof that this surgical approach is successful in treating synkinesis and improving the quality of smiles. The authors concluded that the combination of myectomy and neurectomy, along with functioning free muscle transplantation, offers hopeful and enduring outcomes for synkinetic patients with type II and III, even though there may be a significant need for revisions [44]. Moreover, according to Bran et al. Selective neurolysis seems to be a highly effective alternative approach for treating facial nerve syndrome, a condition that greatly affects one's quality of life due to movement disorders when there is no therapeutic improvement observed with Botox A [45]. To comprehend the treatment approaches, it is crucial to have a fundamental comprehension of synkinesis' underlying mechanisms. Assessing the synkinetic symptoms and individual patterns is necessary via standardized procedures. Initially, facial training is the preferred treatment, followed by the utilization of botulinum toxin. Surgical intervention is only considered for individual cases that do not respond adequately to the initial treatment options [46].

## Conclusions

Selective neurectomy offers a promising surgical solution for the treatment of nonflaccid facial paralysis (NFFP) and synkinesis, both of which can significantly impact the quality of life of affected individuals. This procedure aims to address involuntary movements and hypertonicity by selectively severing problematic nerve-muscle connections, leading to improvements in overall facial function and appearance. While existing evidence indicates positive outcomes, further research is required to comprehensively understand the long-term effects and potential complications associated with selective neurectomy. Furthermore, modified versions of the procedure and combination approaches show potential in treating synkinesis. Given the complexity of facial paralysis and the unique needs of each patient, treatment plans should be customized to individual requirements, considering factors like timing and the type of treatment.
